# Supplementary material for: Single-cell transcriptomics reveals common epithelial response patterns in human acute kidney injury
Source: Genome Med. 2022 Sep 9;14:103. doi: 10.1186/s13073-022-01108-9 (PMC9462075; doi:10.1186/s13073-022-01108-9)
Supplement: Supplementary file 1 — Additional file 1: Fig. S1. Basic statistics of snRNA-seq libraries. Fig. S2. PCA analyses of different cell types and whole tissue. Fig. S3. In situ staining for IGFBP7. Fig. S4. Number of differentially expressed genes between COVID AKI and non-COVID AKI. Fig. S5. Subclustering of leukocytes. Fig. S6. Abundances and marker genes from subclusterings of major cell types. Fig. S7. QC metrics for all subclusters. Fig. S8. Cluster abundances per cell type and sample. Fig. S9. Results from cross-species approach for human PT cells. Fig. S10-12. Subclusterings of remaining kidney epithelial cell types. Fig. S13. Joint heatmap for marker genes of the “New” clusters. Fig. S14. Validation of “New” clusters in PT and TAL cells in an independent dataset. [file 13073_2022_1108_MOESM1_ESM.docx]

Supplementary Material for

**Single-cell transcriptomics reveals common epithelial response patterns in human acute kidney injury**

Christian Hinze^1,2,3^, Christine Kocks^4^, Janna Leiz^2,3^, Nikos Karaiskos^4^, Anastasiya Boltengagen^4^, Shuang Cao^1,2,3^, Christopher Mark Skopnik^2,5^, Jan Klocke^2,5,6^, Jan-Hendrik Hardenberg^2^, Helena Stockmann^2^, Inka Gotthardt^2^, Benedikt Obermayer^7^, Laleh Haghverdi^4^, Emanuel Wyler^4^, Markus Landthaler^4^, Sebastian Bachmann^8^, Andreas C. Hocke^6,9^, Victor Corman^10^, Jonas Busch^11^, Wolfgang Schneider^12^, Nina Himmerkus^13^, Markus Bleich^13^, Kai-Uwe Eckardt^2,*^, Philipp Enghard^2,5*^, Nikolaus Rajewsky^4,*^ Kai M. Schmidt-Ott^1,2,3,*,#^

1 Department of Nephrology and Hypertension, Hannover Medical School, Hannover, Germany.

2 Department of Nephrology and Medical Intensive Care, Charité-Universitätsmedizin Berlin, Corporate Member of Freie Universität Berlin and Humboldt-Universität zu Berlin, Berlin, Germany.

3 Max Delbrueck Center for Molecular Medicine in the Helmholtz Association, Berlin, Germany.

4 Berlin Institute for Medical Systems Biology, Max Delbrueck Center in the Helmholtz Association, Berlin, Germany.

5 Deutsches Rheumaforschungszentrum, an Institute of the Leibniz Foundation, Berlin, Germany.

6 Berlin Institute of Health, Berlin, Germany.

7 Core Unit Bioinformatics, BIH/Charité/MDC, Berlin, Germany.

8 Institute for Functional Anatomy, Charité-Universitätsmedizin Berlin, Corporate Member of Freie Universität Berlin and Humboldt-Universität zu Berlin, Berlin, Germany.

9 Department of Infectious Diseases and Respiratory Medicine, Charité-Universitätsmedizin Berlin, Corporate Member of Freie Universität Berlin and Humboldt-Universität zu Berlin, Berlin, Germany.

10 Institute of Virology, Charité-Universitätsmedizin, Berlin, Germany.

11 Department of Urology, Charité-Universitätsmedizin Berlin, Corporate Member of Freie Universität Berlin and Humboldt-Universität zu Berlin, Berlin, Germany.

12 Department of Pathology, Charité-Universitätsmedizin Berlin, Corporate Member of Freie Universität Berlin and Humboldt-Universität zu Berlin, Berlin, Germany.

13 Institute of Physiology, Christian-Albrechts-Universität, Kiel, Germany.

* Senior authors. # Corresponding author. Email: [schmidt-ott.kai@mh-hannover.de](mailto:schmidt-ott.kai@mh-hannover.de)

Key words: Acute kidney injury, critical illness, single-cell sequencing

**
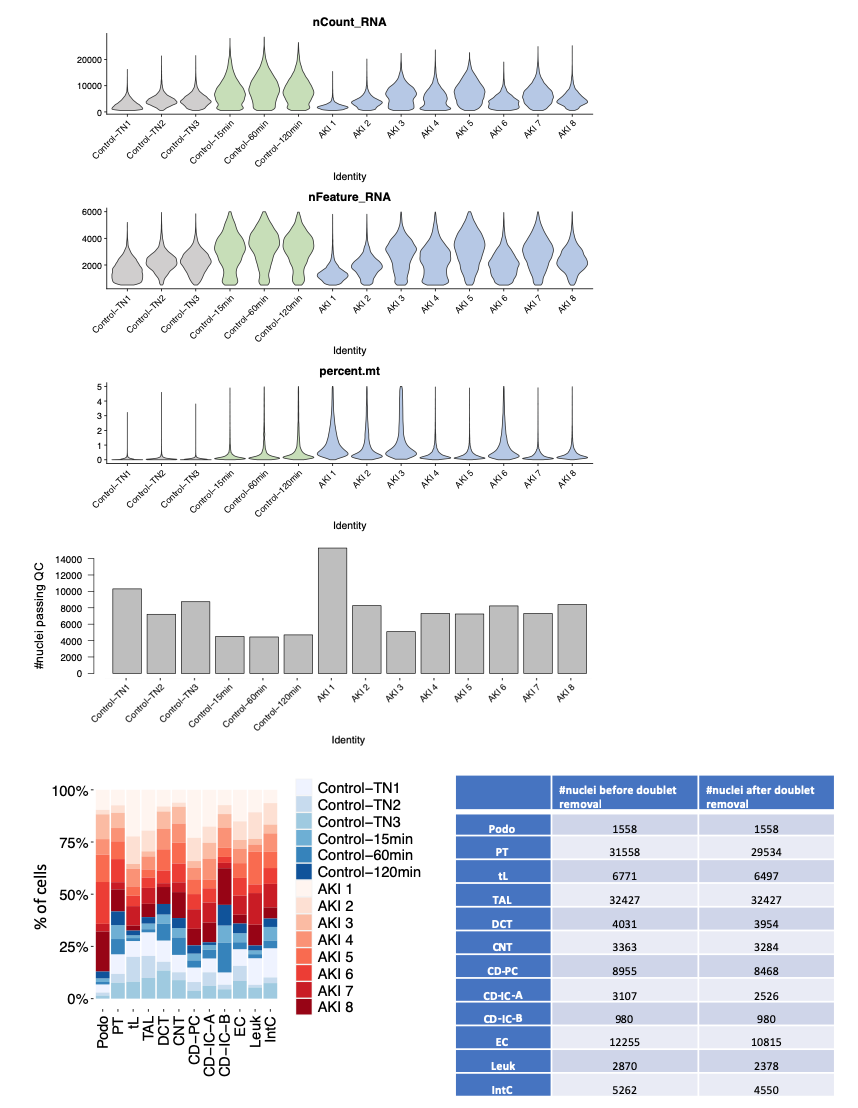
**

***Figure S1: Basic statistics of snRNA-seq libraries.*** *Displayed are plots for number of detected transcripts (nCount_RNA), genes (nFeature_RNA), percent mitochondrial reads (percent.mt) and the number of nuclei which passed quality control per sample. The lowest plots display the relative abundance of samples within major cell types (left) and the number of nuclei before and after doublet removal.*

***Figure S2:*** *PCA analyses analogous to Fig. 1F using cell type-specific highly variable genes for the remaining cell types and whole tissue.*


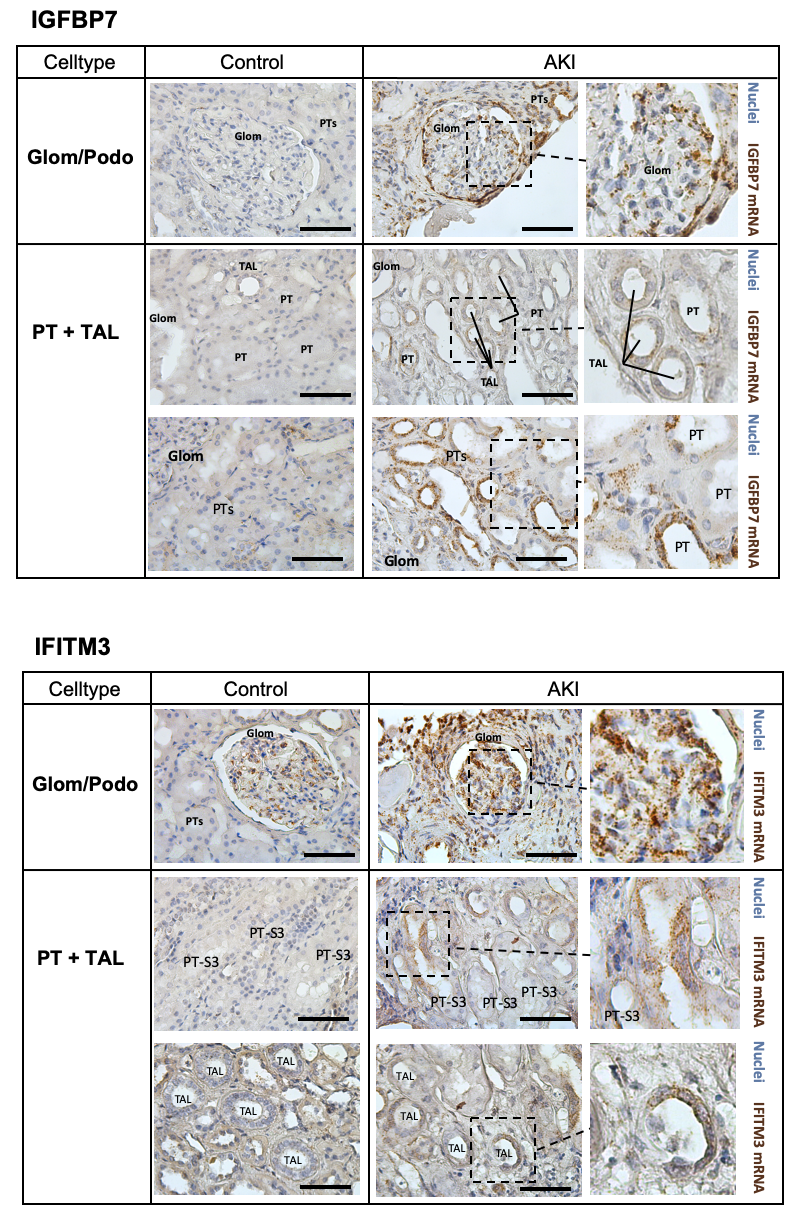


***Figure S3:*** *RNAscope in situ hybridizations of injury insulin-like growth factor binding protein 7 (IGFBP7) and interferon-induced transmembrane protein 3 (IFITM3) on post mortem control and AKI sample. Scale bar: 50µm.*

**
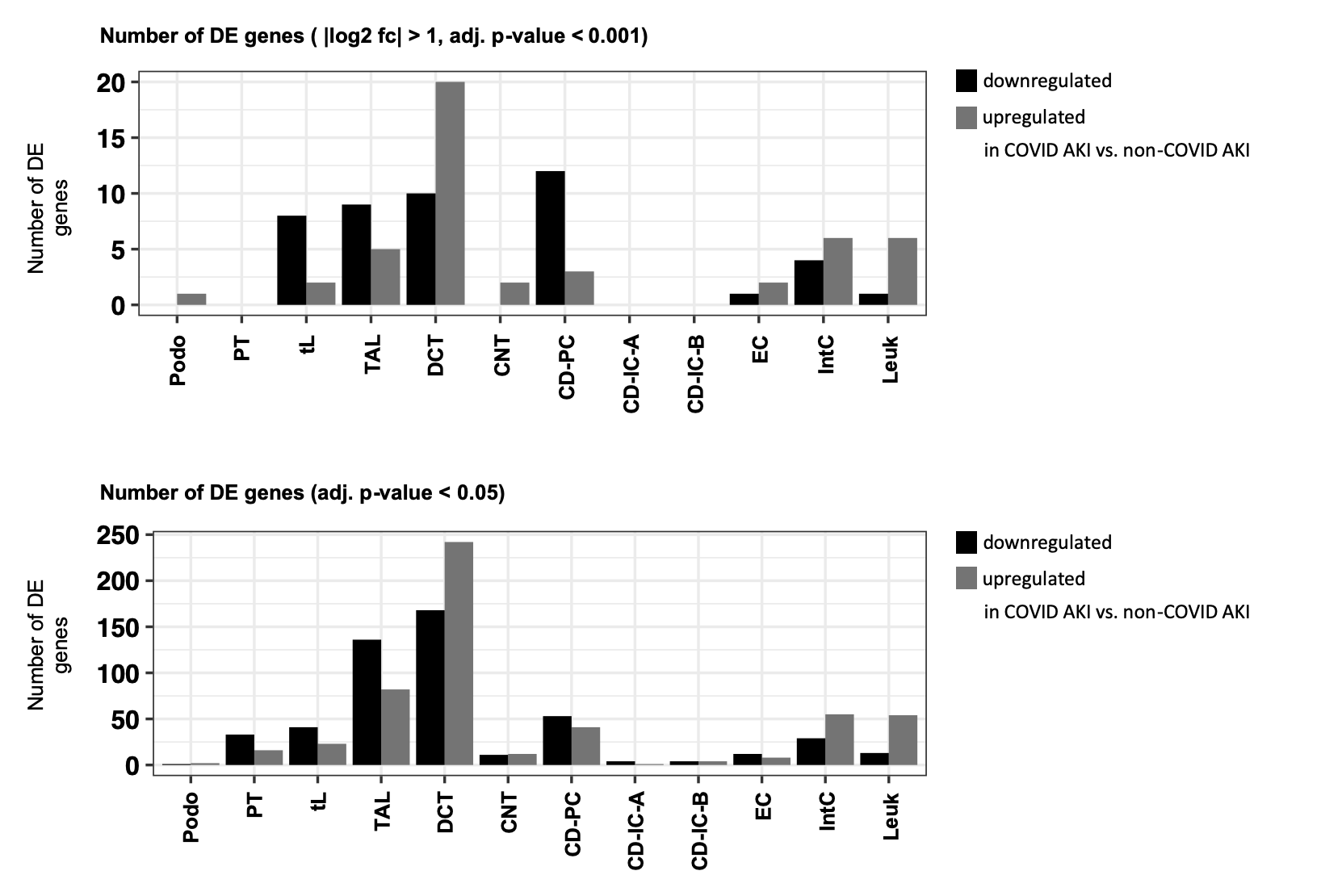
**

***Figure S4: Number of differentially expressed genes between COVID AKI and non-COVID AKI.*** *The upper bar plot depicts the number of differentially expressed genes between COVID AKI and non-COVID AKI applying the same cut off values for fold change (absolute log2 FC >1) and adjusted p-value (<0.001) as in the comparison between AKI and control samples. These cut off values were chosen to be rather strict to extract the most significant signals from the strong AKI versus control signal. The lower plot displays the number of differentially expressed genes between COVID AKI and non-COVID AKI by applying less stringent criteria (adjusted p-value cut off of 0.05). Please refer to Suppl. Table S5 for a list of differentially expressed genes.*

*
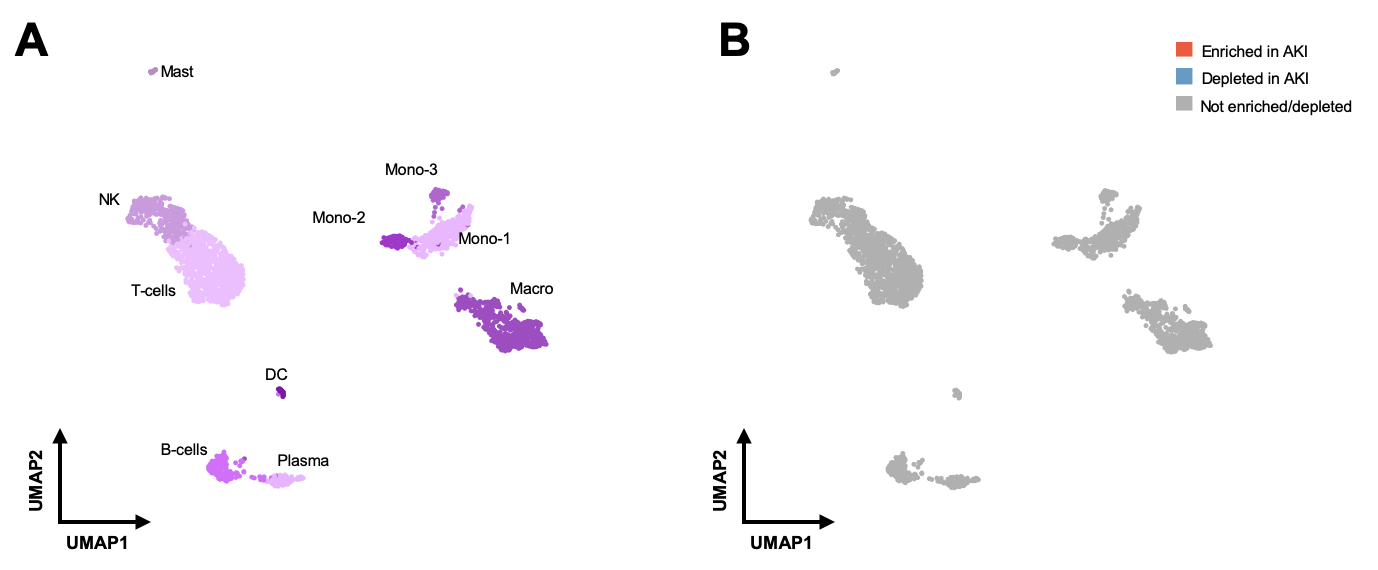
*

***Figure S5: Subclustering of leukocytes. A, B.*** *UMAP plot of subclustered leukocytes (A) and their enrichment or depletion in AKI based on statistical testing of relative abundances within the leukocytes (B) (see methods for details). In B, the same UMAP plot as in A is color-coded based on enrichment (red) or depletion (blue) in AKI individuals. Mast – mast cells, DC – dendritic cells, NK – natural killer cells, Plasma – plasma cells, Mono – monocytes, Macro – macrophages*

**
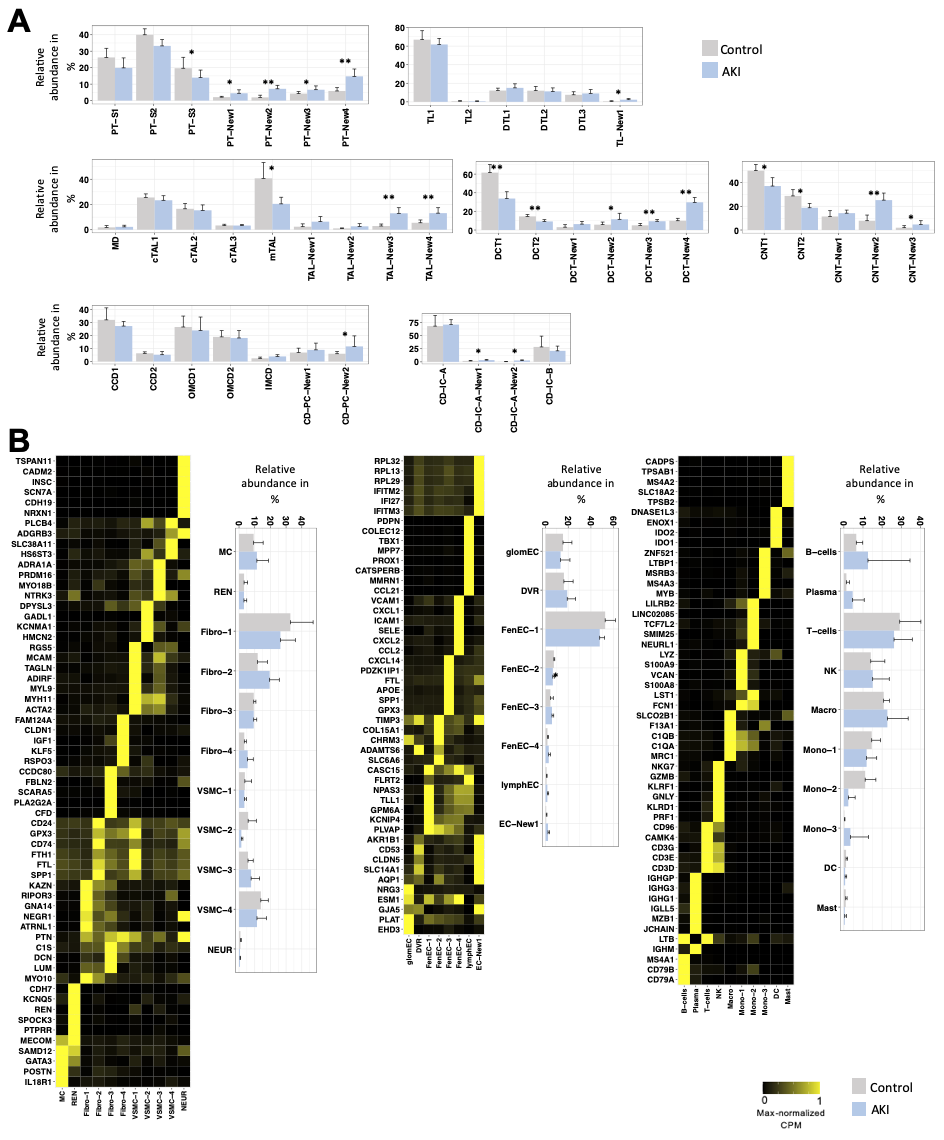
**

***Figure S6: Abundances and marker gene expression of cell populations from subclusterings. A.*** *Relative abundances per major cell type (CD-IC-A and CD-IC-B were analyzed together as CD-IC) of renal cell subpopulations from subclustering analyses of kidney epithelial cells.* ***B.*** *Heatmaps with marker genes and abundance plots as in (A) for ECs, IntCs and leukocytes. Note that marker gene heatmaps for kidney epithelial cell subclusters are shown in figures 4, 5 and Suppl. Fig. S7-9. PT-S1-3 – PT S1-3 segments, c/mTAL – cortical/medullary TAL, TL, DTL – thin limb, descending thin limb, CCD, OMCD, IMCD – cortical/outer and inner medullary collecting duct principal cell; lymphEC – lymphatic EC, GEC – glomerular EC, FenEC 1-4 – fenestrated endothelial cell types, DVR – descending vasa recta; MC – mesangial cells, VSMC – vascular smooth muscle cells, REN – renin-transcribing cells, Fibro – fibroblasts, NEUR – neuronal cells; Mast – mast cells, DC – dendritic cells, NK – natural killer cells, Plasma – plasma cells, Mono – monocytes, Macro – macrophages.*

******

***Figure S7:*** *Quality control metrics and cell numbers for all identified subclusters.*

*
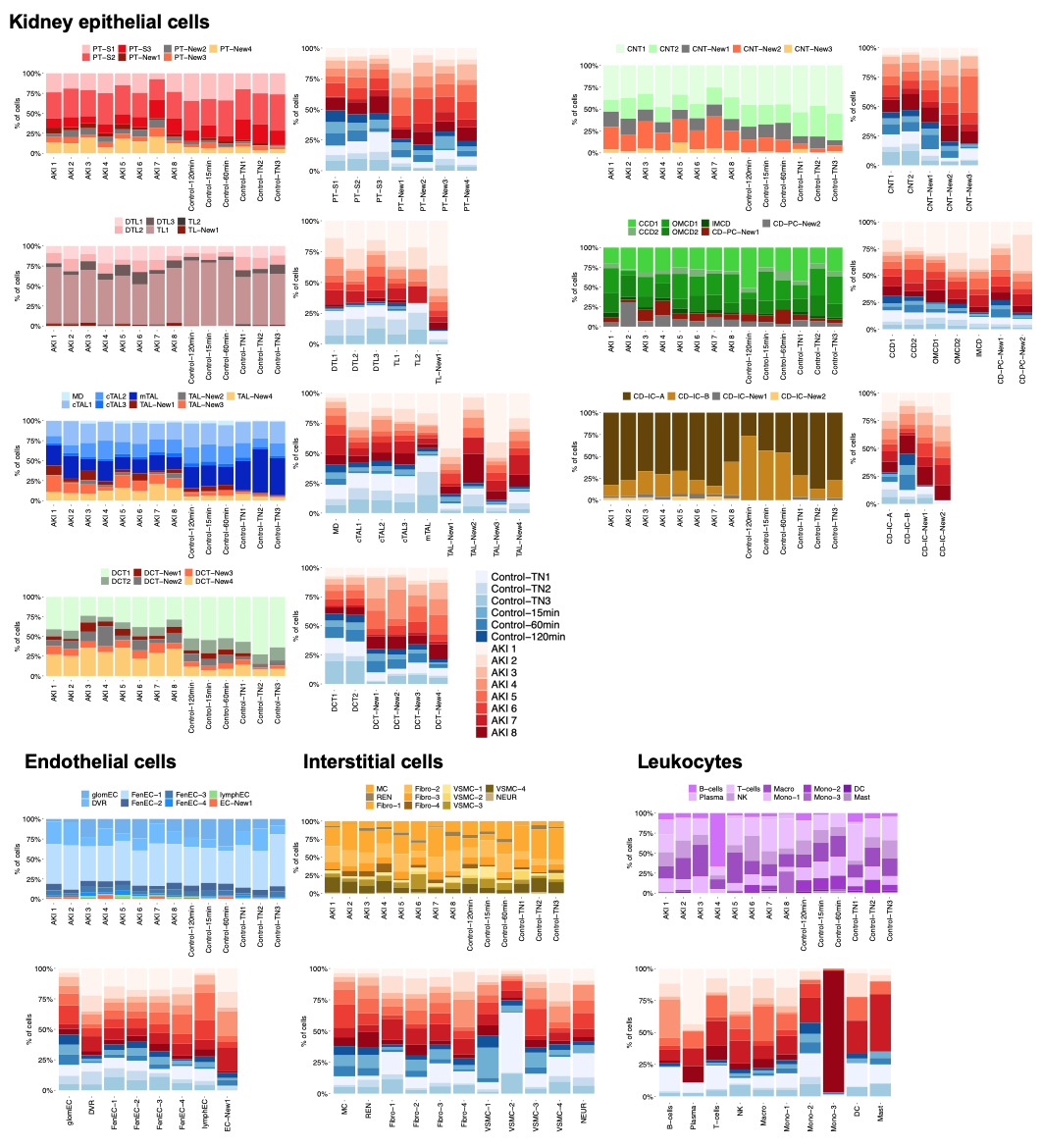
*

***Figure S8:*** *Bar plots with relative abundances per sample and sub cell type for kidney epithelial cells, endothelial cells, interstitial cells and leukocytes.*

**
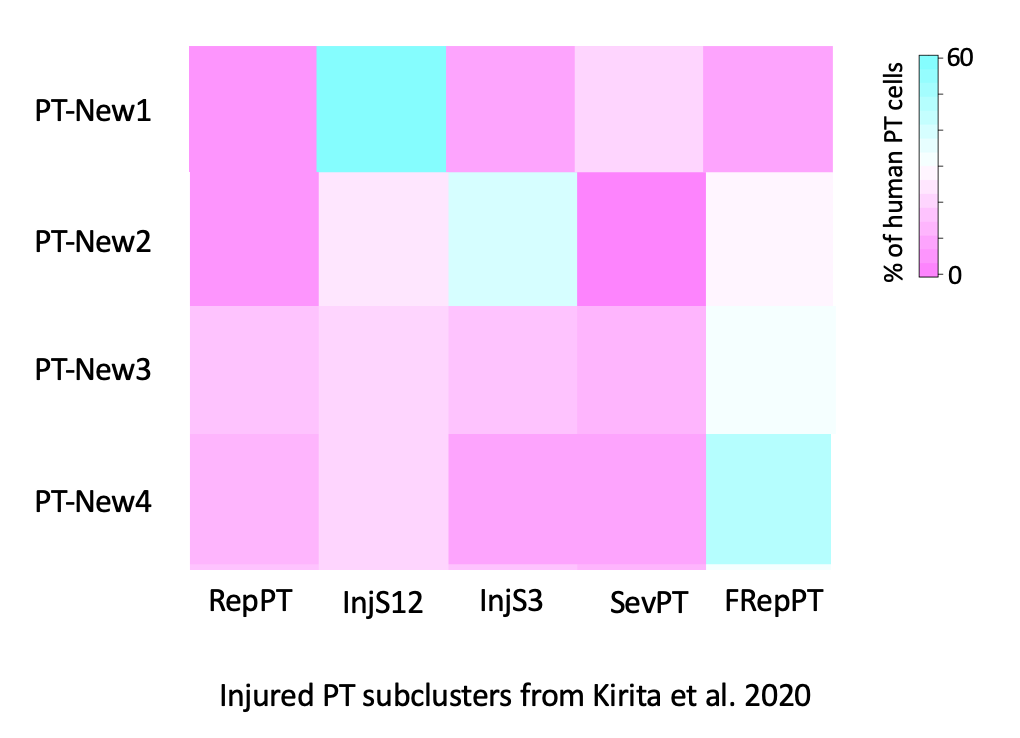
**

***Figure S9:*** *Results from cross-species approach using published mouse ischemia reperfusion-induced AKI data^1^. The color code indicates the percentage of PT cells from PT-New1-4 in our snRNA-seq data which were assigned to the indicated mouse PT clusters. Abbreviations from the original publication: RepPT – repairing PT, InjS12 – injured S1/2 PT segments, InjS3 – injured S3 segment, SevPT – severely injured PT, FRepPT – failed repair PT.*

**
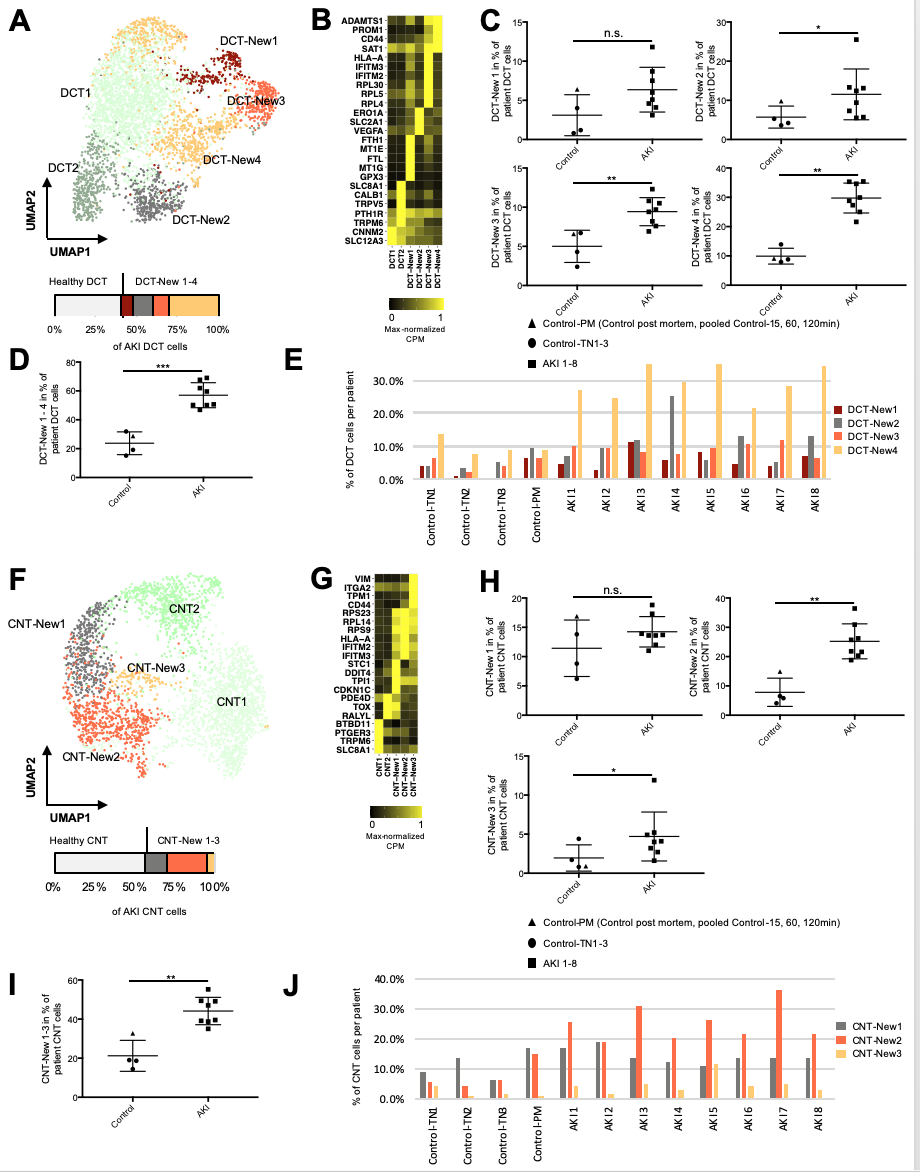
**

***Figure S10: A.*** *UMAP plot of subclustering of the DCT. Below the UMAP is a bar plot displaying the relative abundances of DCT-New 1-4 with respect to all AKI DCT cells.* ***B.*** *Heatmap of selected marker genes for the identified DCT cell subpopulations.* ***C.*** *Plots displaying relative abundances of DCT-New 1-4 with respect to the individual’s DCT cells, separately.*  ***D.*** *Relative abundances of combined DCT-New 1-4 with respect to the individual’s DCT cells.* ***E.*** *Individual abundances of DCT-New 1-4 for control and AKI individuals.* *P-value: *<0.05, **<0.01, ***<0.001, n.s. – not significant. Control-PM – pooled samples (*Control_15min_, Control_60min_, Control_120min_*) of post mortem non-AKI control individual.*

**
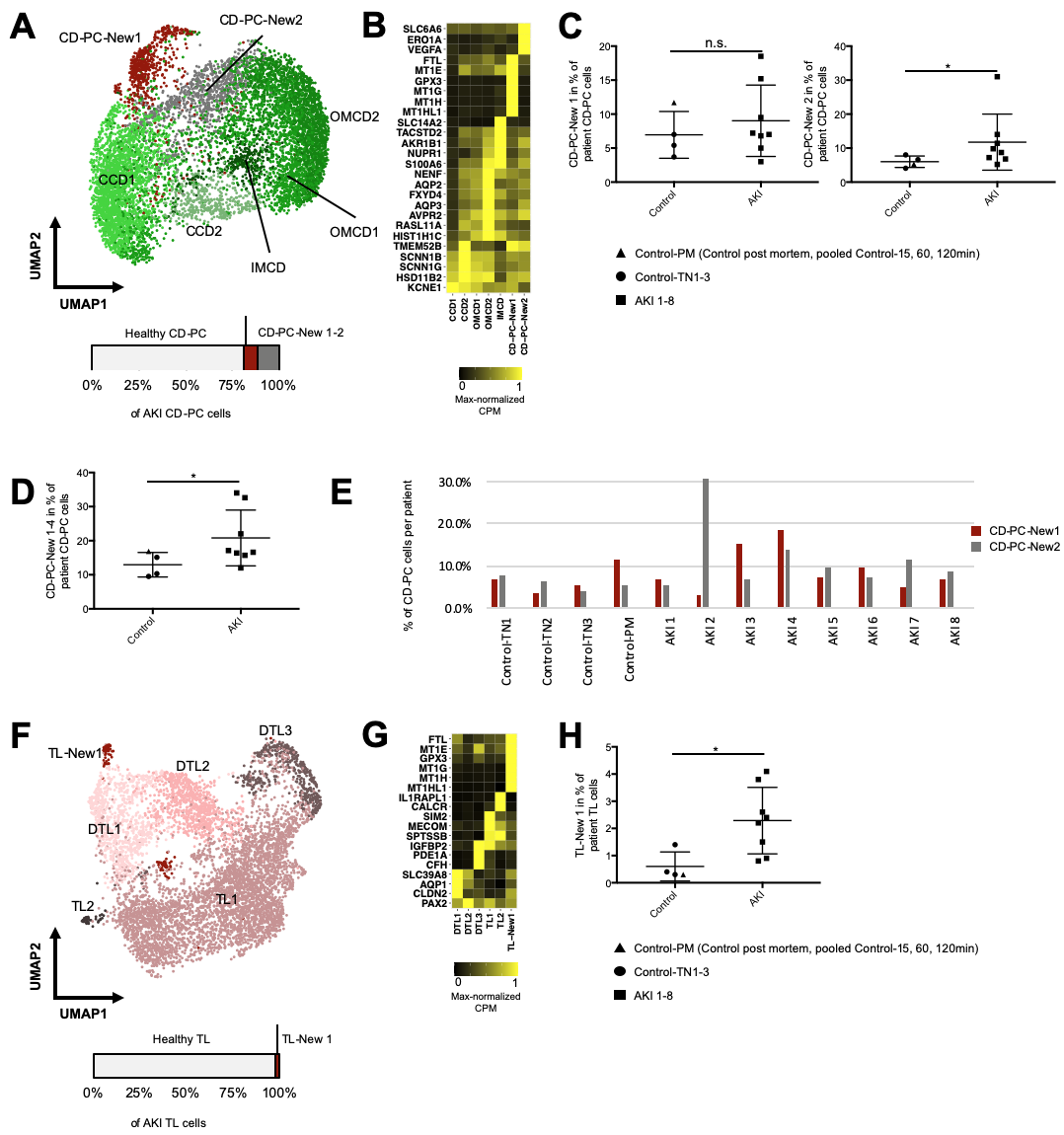
**

***Figure S11:*** *Analogous figure as shown in Suppl. Fig. S6 for CD-PC (A-E) and thin limbs (F-J). Note that for the thin limbs in F, DTL clusters 1-3, no ascending TL clusters and clusters only named TL1, 2 are depicted. The reason for this is (compare to CD-PC) that we expect very little ascending thin limb cells in our data. The TL clusters TL1 and 2 express SPTSSB, which is an ascending thin limb marker gene, but only little CLCNKA. TL1 and TL2 are very abundant compared to the DTL clusters and are therefore unlikely to be clean ascending thin limb clusters. Cells with characteristics as TL1 and 2 (expression of SPTSSB, little expression of AQP1 and CLCNKA) were described in mouse kidneys before^2^.*


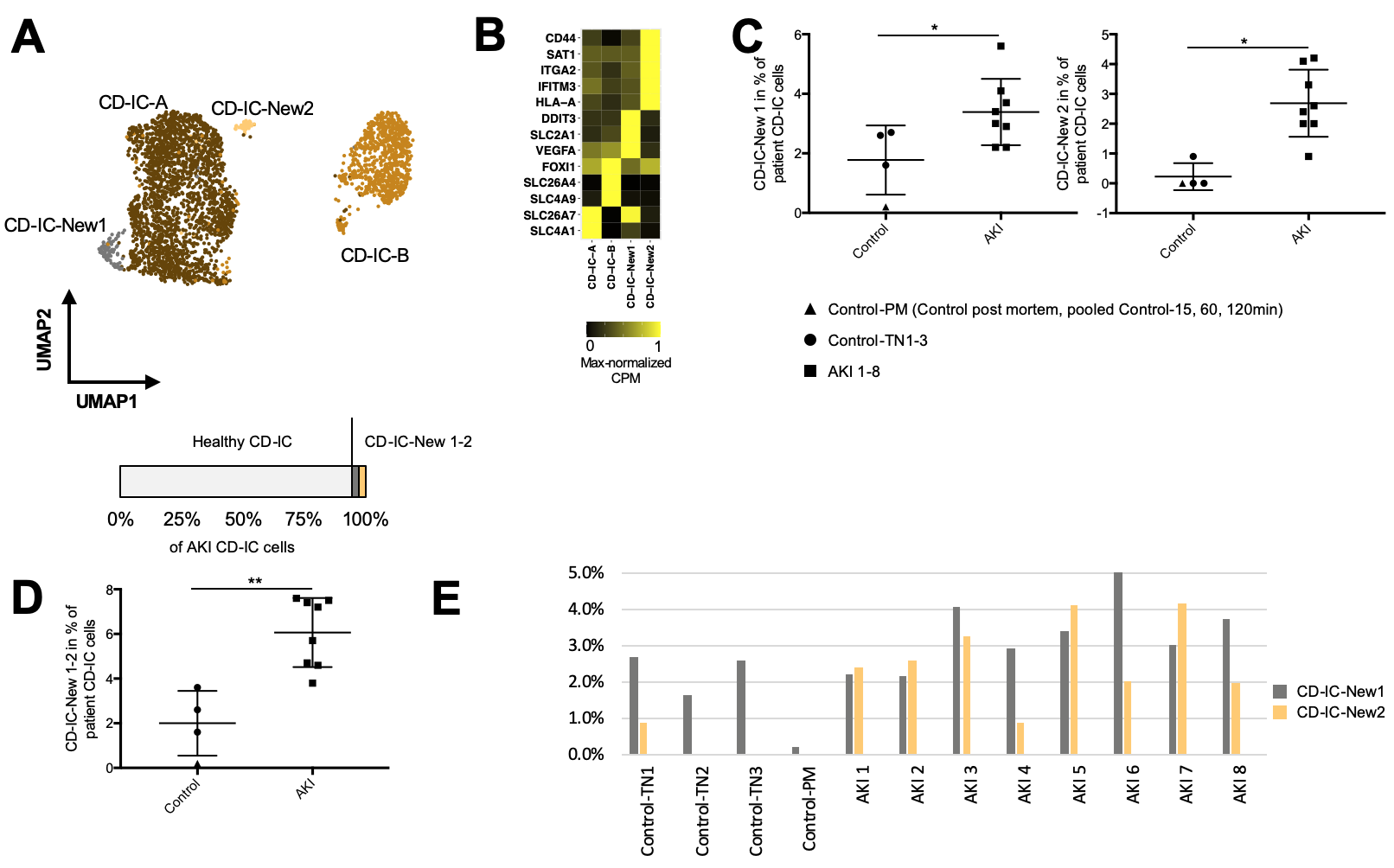


***Figure S12:*** *Analogous figure as shown in Suppl. Fig. S6 for CD-IC.*

**
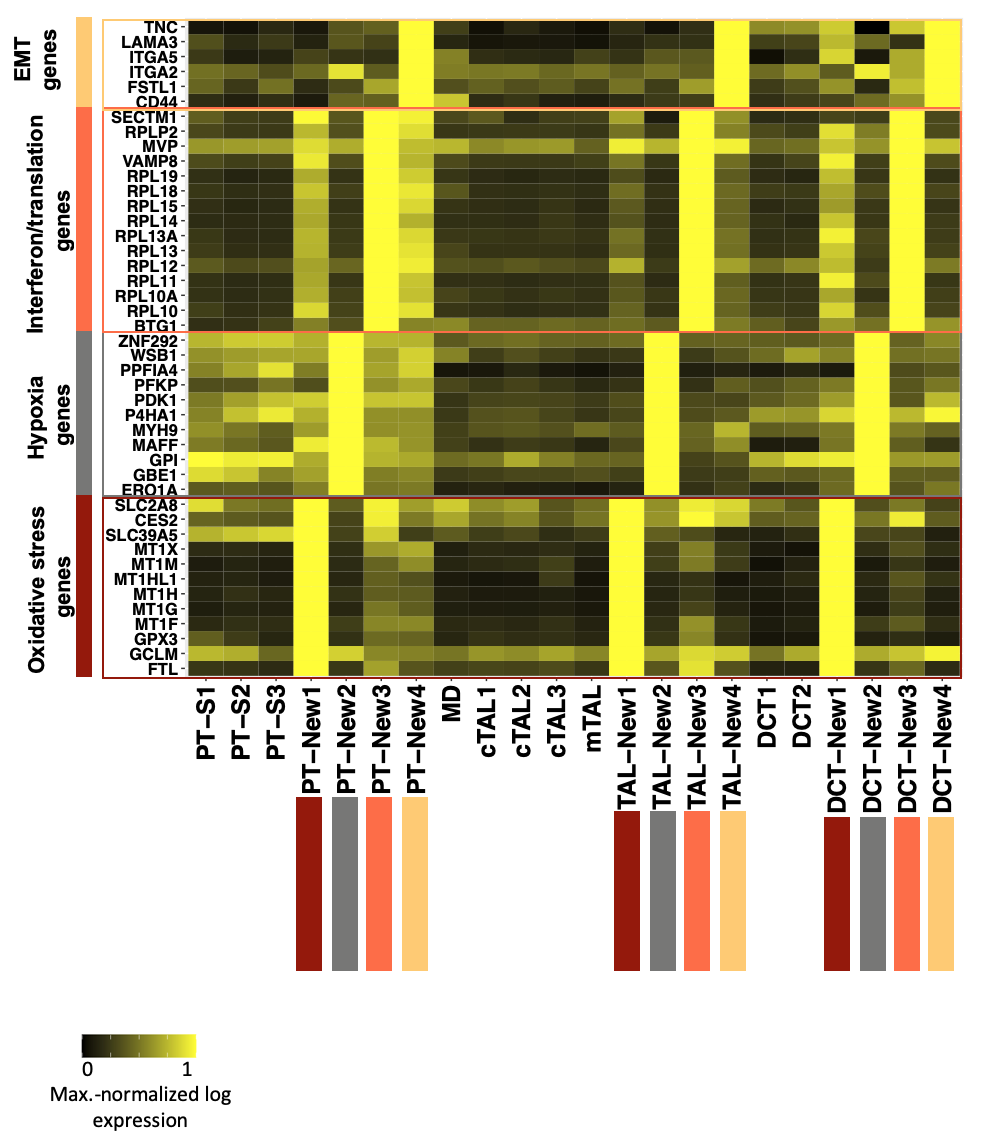
**

***Figure S13:*** *Heatmap displaying marker gene overlap between the “New” clusters in the cell types of the kidney tubule which display all four “New” clusters. Color codes indicate the assignment of genes and clusters to the respective gene expression profiles (see also Fig. 4-7, Additional file 1: Suppl. Fig. 10-12). Log average gene expression values per cluster were maximum-normalized per cell type. Genes were selected based on the gene lists presented in Additional file 8: Table S7.*

***
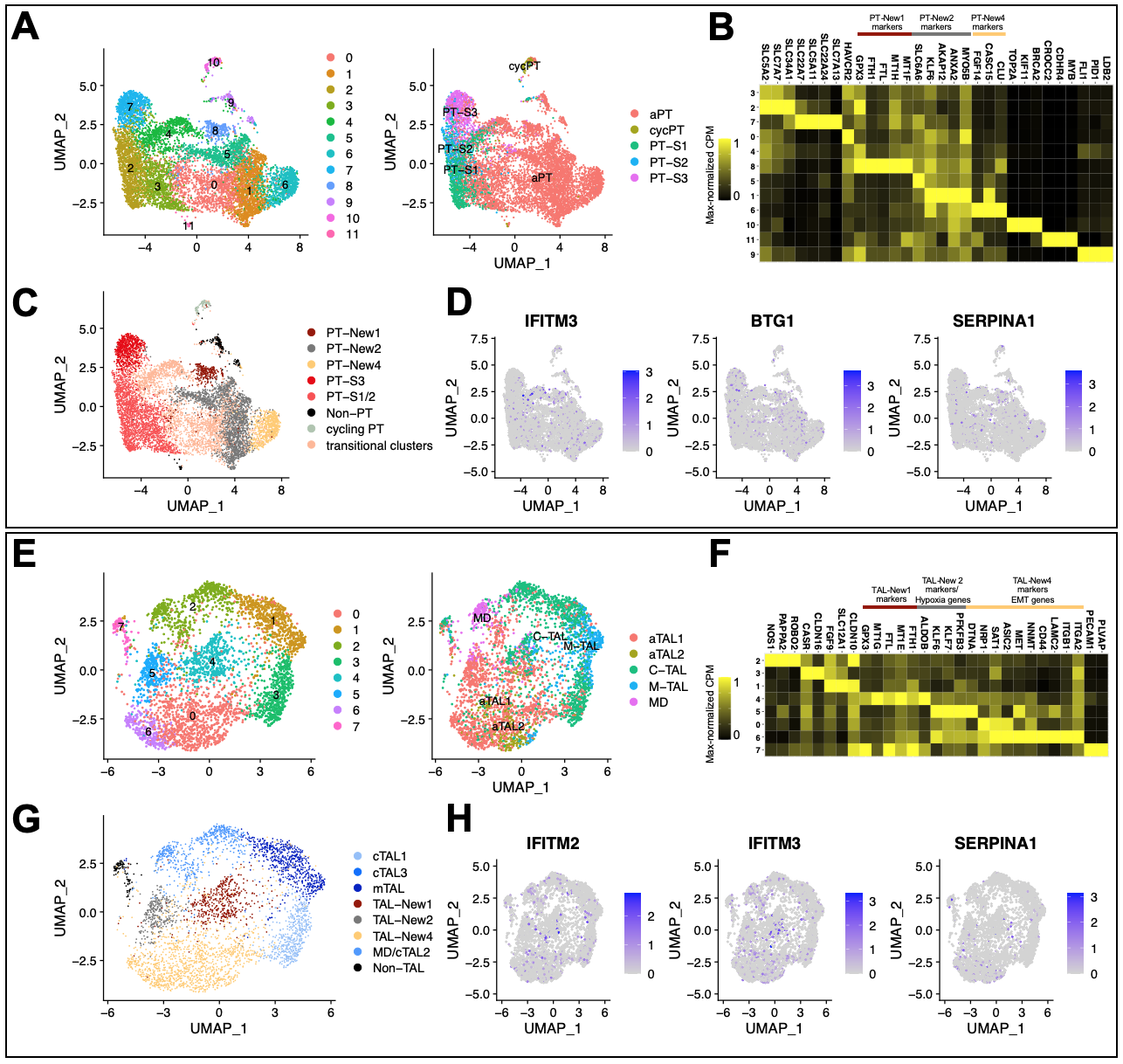
***

***Figure S14: Validation of PT-New and TAL-New clusters in an independent dataset (Lake et al.^3^). A.*** *Unbiased clustering (left panel) of PT cells from Lake et al. On the right panel, the cluster labeling from the original publication is highlighted (aPT – adaptive PT, cycPT – cycling PT).* ***B.*** *Heatmap showing marked overlap of marker gene expression between Lake et al. PT clusters PT-New 1, 2 and 4 marker genes (Fig. 4, Additional file 7: Table S6).* ***C.*** *Based on marker gene expression, PT cells from Lake et al. have been colored and labeled according to the PT nomenclature of the data from this manuscript (Fig. 4).* ***D.*** *Feature plot on PT cells from Lake et al. for inflammation-associated PT-New 3 marker genes IFITM3, BTG1 (both interferon target genes) and SERPINA1 showing only very sparse and scattered expression.* ***E.-H.*** *Analogous panels for TAL cells from Lake et al.*

**References**

1. Kirita, Y., Wu, H., Uchimura, K., Wilson, P.C. & Humphreys, B.D. Cell profiling of mouse acute kidney injury reveals conserved cellular responses to injury. *Proc Natl Acad Sci U S A* **117**, 15874-15883 (2020).

2. Ransick, A.*, et al.* Single-Cell Profiling Reveals Sex, Lineage, and Regional Diversity in the Mouse Kidney. *Dev Cell* **51**, 399-413 e397 (2019).

3. Lake, B.B.*, et al.* An atlas of healthy and injured cell states and niches in the human kidney. *bioRxiv*, 2021.2007.2028.454201 (2021).
